# Nesfatin-1/Nucleobindin-2 enhances cell migration, invasion, and epithelial-mesenchymal transition via LKB1/AMPK/TORC1/ZEB1 pathways in colon cancer

**DOI:** 10.18632/oncotarget.9140

**Published:** 2016-05-02

**Authors:** Jung-Yu Kan, Meng-Chi Yen, Jaw-Yuan Wang, Deng-Chyang Wu, Yen-Jung Chiu, Ya-Wen Ho, Po-Lin Kuo

**Affiliations:** ^1^ Graduate Institute of Clinical Medicine, College of Medicine, Kaohsiung Medical University, Kaohsiung, Taiwan; ^2^ Division of Gastrointestinal and General Surgery, Department of Surgery, Kaohsiung Medical University Hospital, Kaohsiung, Taiwan; ^3^ Department of Emergency Medicine, Kaohsiung Medical University Hospital, Kaohsiung Medical University, Kaohsiung, Taiwan; ^4^ Center for Biomarkers and Biotech Drugs, Kaohsiung Medical University, Kaohsiung, Taiwan; ^5^ Division of Gastroenterology, Department of Internal Medicine, Kaohsiung Medical University Hospital, Kaohsiung, Taiwan; ^6^ Institute of Medical Science and Technology, National Sun Yat-Sen University, Kaohsiung, Taiwan

**Keywords:** Nucleobindin-2 (NUCB-2), nesfatin-1, colon cancer, EMT, metastasis

## Abstract

Recent studies indicate that a high level of nesfatin-1/Nucleobindin-2 (NUCB-2) is associated with poor outcome and promotes cell migration in breast cancer and prostate cancer. However, the role of NUCB2 is not well known in colon cancer. In this study, NUCB-2 level in colon cancer tissue was higher than that in non-tumor tissue. Suppression of NUCB-2 in a colon cancer cell line SW620 inhibited migration and invasion. The microarray analysis showed that low expression level of transcription factor ZEB1 in NUCB-2 knockdowned SW620 cells. In addition, expression level of epithelial-mesenchymal transition (EMT)-related molecules including N-cadherin, E-cadherin, β-catenin, Slug and Twist was affected by NUCB-2 suppression and ZEB1-denepdent pathway. The signaling pathway liver kinase B1(LKB1)/AMP-dependent protein kinase (AMPK)/target of rapamycin complex (TORC) 1 was involved in regulation of NUCB-2-mediated metastasis and EMT properties. Suppression of NUCB-2 inhibited tumor nodules formation in a murine colon tumor model as well. In summary, nesfatin-1/NUCB-2 enhanced migration, invasion and EMT in colon cancer cells through LKB1/AMPK/TORC1/ZEB1 pathways *in vitro* and *in vivo*.

## INTRODUCTION

Colorectal cancer (also known colon cancer) is the third most common cancer type and the third cause of cancer-related deaths worldwide [1]. According to cancer statistics, approximately 20–25% of patients with colon cancer have metastases at the time of diagnosis and 50–60% of the remainder will develop metastatic colon cancer [2, 3]. Currently, cytotoxic chemotherapy is the major treatment for colon cancer [4]. However, the therapeutic effect still needs to be improved. Therefore, investigation of novel regulatory pathways of metastasis in colon cancer is beneficial to developing novel therapeutic strategies.

Nesfatin-1 is a hypothalamic neuropeptide, is derived from its precursor nucleobindin-2 (NUCB-2), and is reported as being an important regulator of energy homeostasis [5, 6]. Distribution of nesfatin-1/NUCB-2 is in the central nervous system, gastrointestinal system, reproductive organs and adipose tissue [7]. Recently, the function of nesfatin-1/NUCB-2 is linked to tumor development and metastasis. High levels of NUCB-2 mRNA and protein is associated with shorter biochemical recurrence-free survival time in prostate cancer [8, 9]. In addition, nesfatin-1 induces cell migration through an autocrine pathway in prostate cancer cells [10]. In breast cancer, NUCB-2 plays an important role in the metastasis of breast cancer cell lines and is a potent prognostic factor for primary breast carcinoma [11]. Furthermore, high NUCB-2 expression is associated with metastasis and shorter overall survival in clear cell renal cell carcinoma tissue [12]. This evidence suggests that NUCB-2 can induce metastasis in some types of cancer. However, nesfatin-1 inhibits cell proliferation in a human adrenocortical carcinoma cell line and an ovarian epithelial carcinoma cell [13, 14]. These controversial results may suggest the regulation of nesfatin-1/NUCB-2 is dependent on tissue specificity.

Nesfatin-1/NUCB-2 is involved in regulation of endocrine system, stress, immune system and cardiovascular system [15]. However, the signaling pathways of nesfatin-1/NUCB-2 are not well known in each tissue. In the brain, nesfatin-1/NUCB-2 inhibits food uptake via a leptin-independent pathway [16]. It increases insulin sensitivity through Akt/AMP-dependent protein kinase (AMPK)/target of rapamycin complex (TORC) 2 pathway in brain [17]. In addition, nesfatin-1 inhibits proliferation of ovarian epithelial carcinoma cells through targeting the rapamycin (mTOR) pathway [14].

To the best of our knowledge, the function and signaling pathways of nesfatin-1/NUCB-2 have not been studied in colon cancer. In order to investigate this issue, we examined the expression levels in serum and tumor tissue of patients and the signaling pathways in colon cancer cell line. Furthermore, we investigated whether nesfatin-1/NUCB-2 affected the tumor development in a murine tumor model and served as a potential biomarker of metastasis in an online microarray dataset.

## RESULTS

### NUCB-2 overexpression is observed in colon tumor tissue

**Figure 1:**
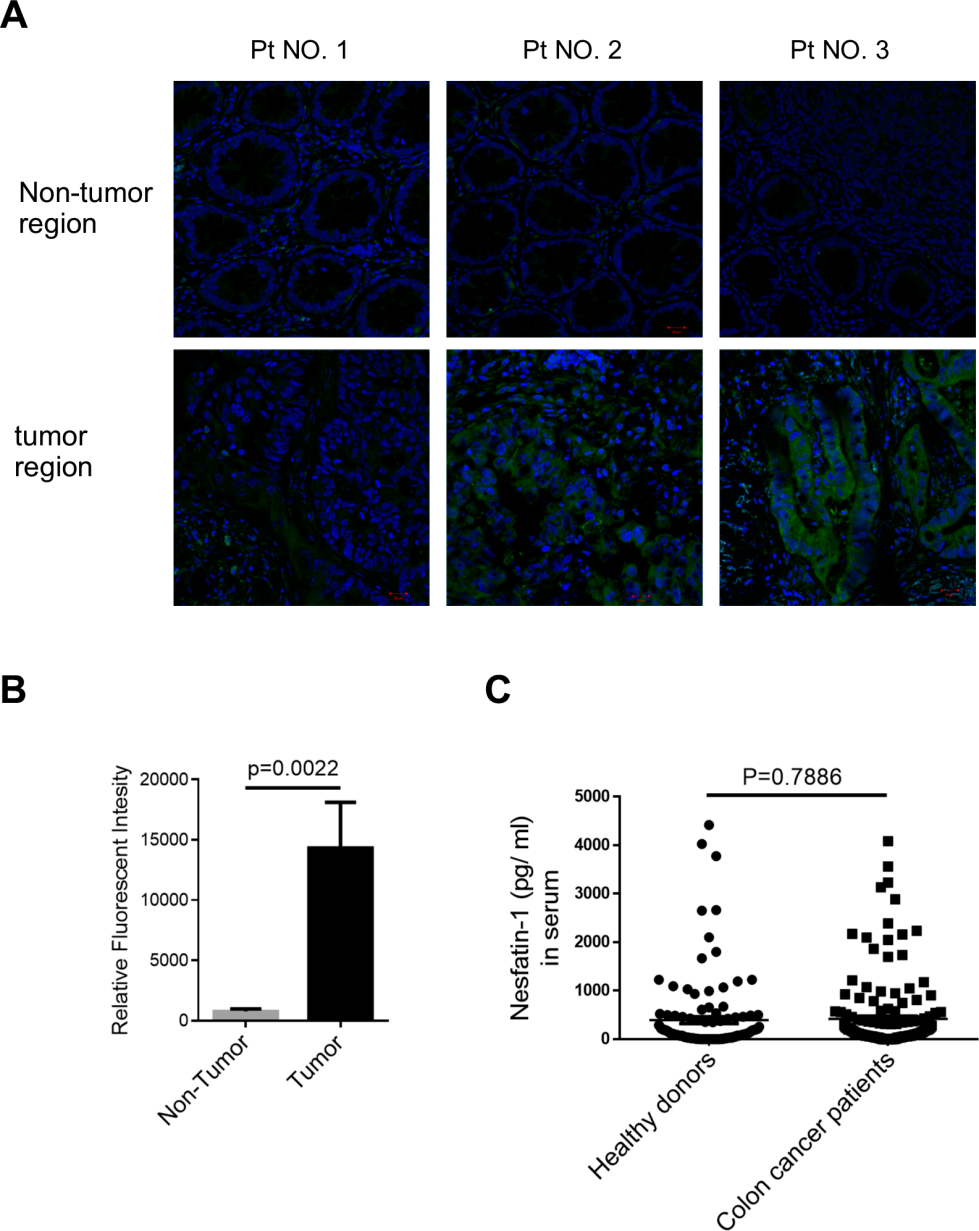
The expression of NUCB-2 in clinical colon cancer (**A**) Immunofluorescent detection of NUCB-2 in frozen section of tumor and non-tumor tissue. Antibody staining is green and nuclei are stained with DAPI. Three of ten pairs were shown. (**B**) Quantification of relative fluorescent intensity in ten pairs. (**C**) Nesfatin-1 concentration in serum of healthy donors (*n* = 119) and colon cancer patients (*n* = 160).

To determine the role of nesfatin-1/NUCB-2 in colon cancer, the NUCB-2 expression was detected in tumor and non-tumor regions in ten pairs of colon cancer samples. The immunofluorescent staining showed the expression NUCB-2 in tumor was higher than that in non-tumor regions (Figure 1A and 1B). In order to further determine whether NUCB-2 overexpression resulted in systematic elevation of NUCB-2, the serum concentration of nesfatin-1, which is derived from N-terminal of NUCB-2, was measured. Our results revealed that there was no significant difference between healthy donors and patients with colon cancer (Figure 1C). It suggests that nesfatin-1/NUCB-2 locally but not systematically regulates signaling pathways of colon cancer cells.

### Suppression of NUCB-2 inhibits migration and invasion in a colon cancer cell line

**Figure 2:**
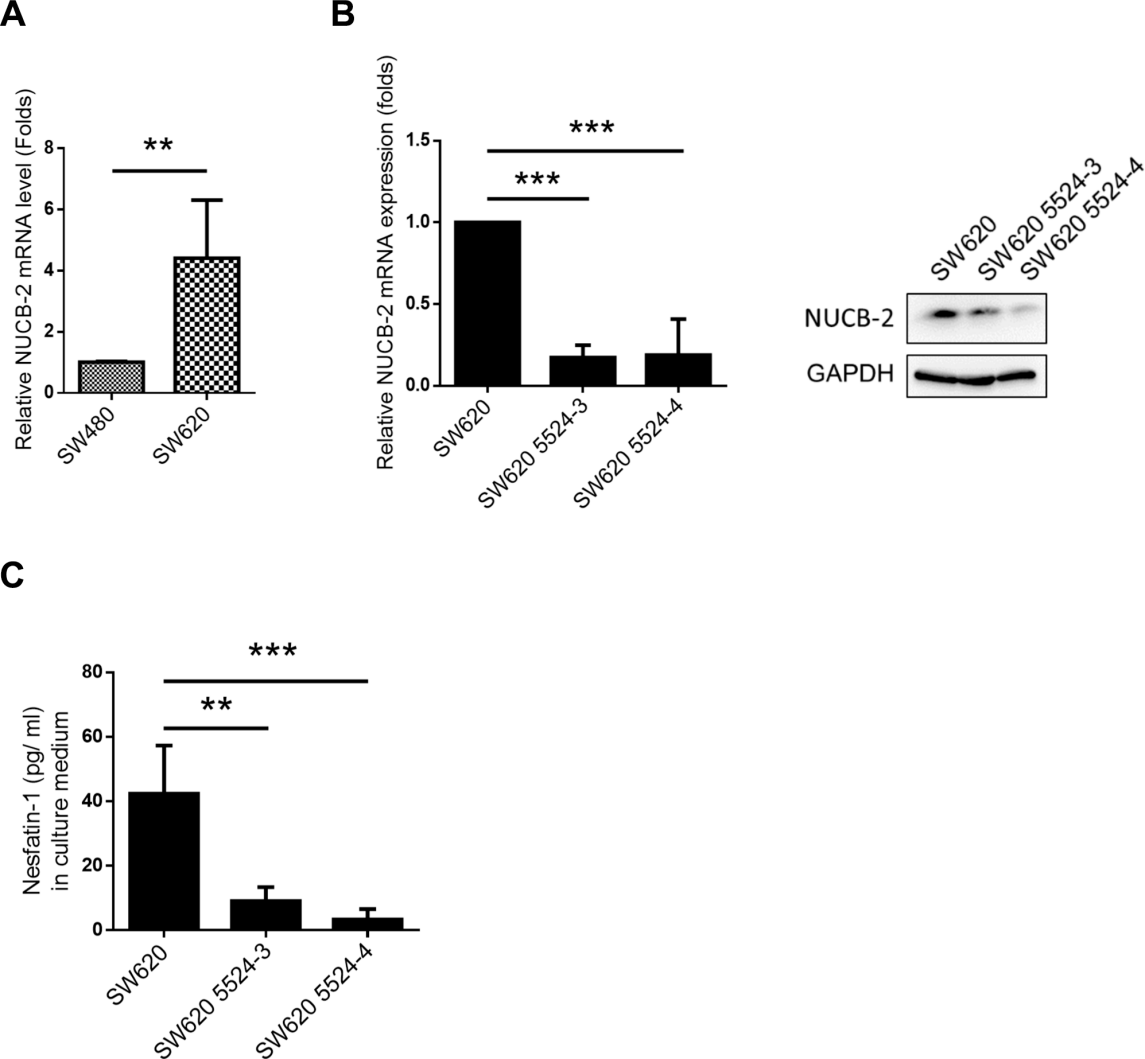
Suppression of NUCB-2 expression in SW620 (**A**) mRNA expression of NUCB-2 in SW480 and SW620. (**B**) Effect of NUCB-2 shRNA. Left panel showed mRNA expression of NUCB-2 in SW620 and NUCB-2-knockdowned stable clones (5524–3 and 5524–4) and right panel showed protein expression of NUCB-2 in SW620 and NUCB-2-knockdowned stable clones. (**C**) Concentration of nesfatin-1 in culture medium of SW620 and NUCB-2-knockdowned stable clones. Data represent mean ± SD. *p < 0.05; **p < 0.01; ***p < 0.001.

**Figure 3:**
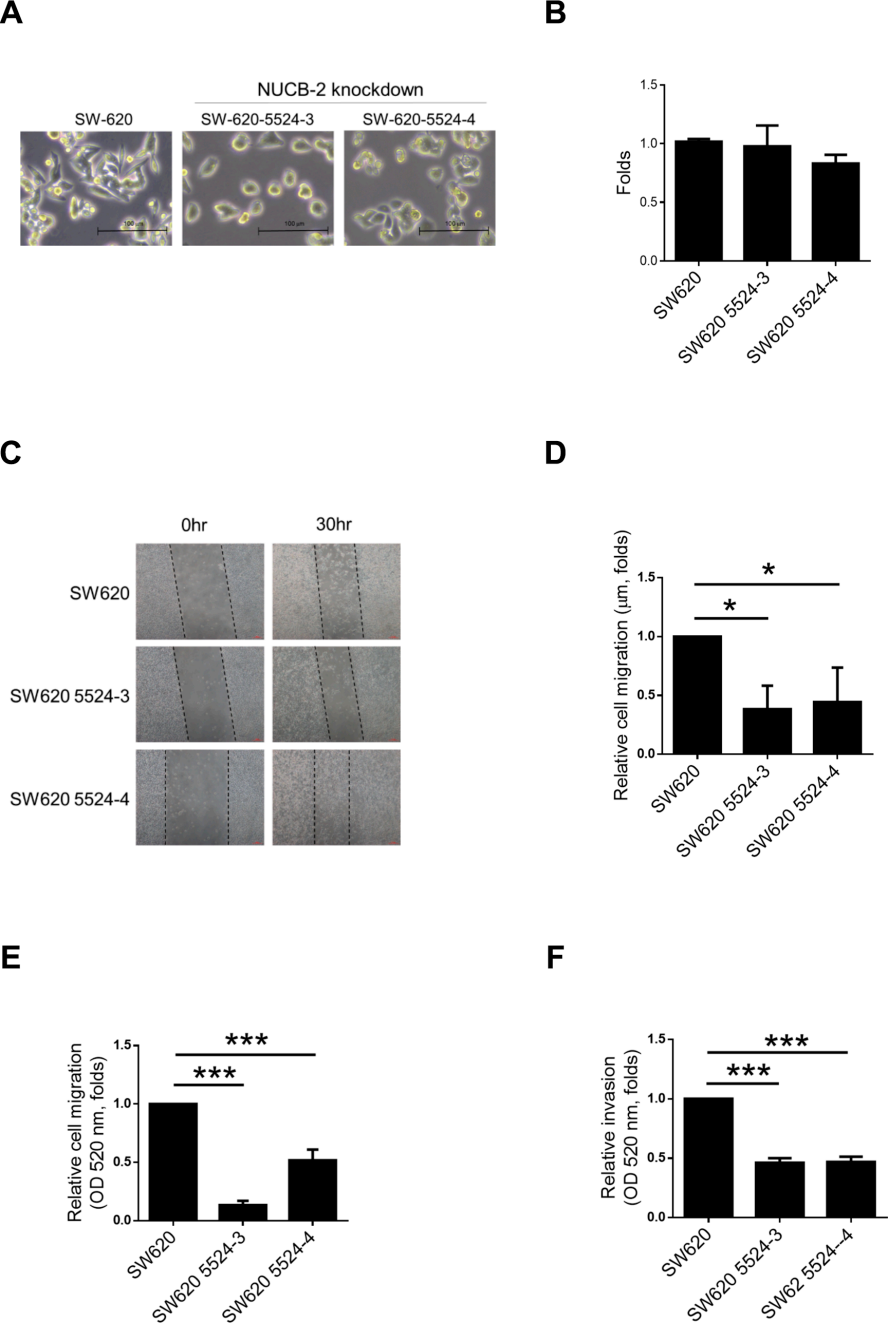
Suppression of NUCB-2 expression inhibited migration and invasion in SW620 (**A**) Morphology of SW620 and NUCB-2-knockdowned stable clones. (**B**) Proliferation assay. (**C**) Wound healing assay. (**D**) Quantitative results of wound healing assay. (**E**). Quantitative results of transwell migration assay. (**F**) Quantitative results of transwell invasion assay. Data represent mean ± SD. *p < 0.05; ***p < 0.001.

SW480 and SW620 are colon cancer cell lines which are derived from the primary colon cancer (SW480) and lymph node metastasis (SW620) of the same patient [18]. SW620 showed higher NUCB-2 expression than SW480 (Figure 2A). Because high NUCB-2 expression was observed in clinical colon tumor samples and SW620 was a metastatic colon cancer cell line, SW620 was used in this study and two NUCB-2 knockdowned stable clones were established (5524-3 and 5524-4). The low levels of mRNA (NUCB-2), endogenous protein (NUCB-2) and secreted protein (nesfatin-1) was detected in both NUCB-2 knockdowned clones (Figure 2B and 2C). Interestingly, suppression of NUCB-2 caused morphological change in SW620. Rounded morphology was observed in NUCB-2 knockdowned cells (Figure 3A). The result implied that NUCB-2 is involved in regulation of epithelial-mesenchymal transition (EMT) properties. The proliferation assay indicated NUCB-2 knockdown did not suppress or enhance proliferation rate in SW620 after 24 hours of incubation (Figure 3B). The wound healing assay showed that migration ability of SW620 was inhibited after NUCB-2 suppression (Figure 3C and 3D). Furthermore, suppression of NUCB-2 reduced the ability of migration and invasion in transwell assay as well (Figure 3E and 3F). It indicates NUCB-2 enhances migration and invasion in colon cancer cells.

### ZEB-1 is critical for regulation of NUCB-2-mediated migration and invasion

**Figure 4:**
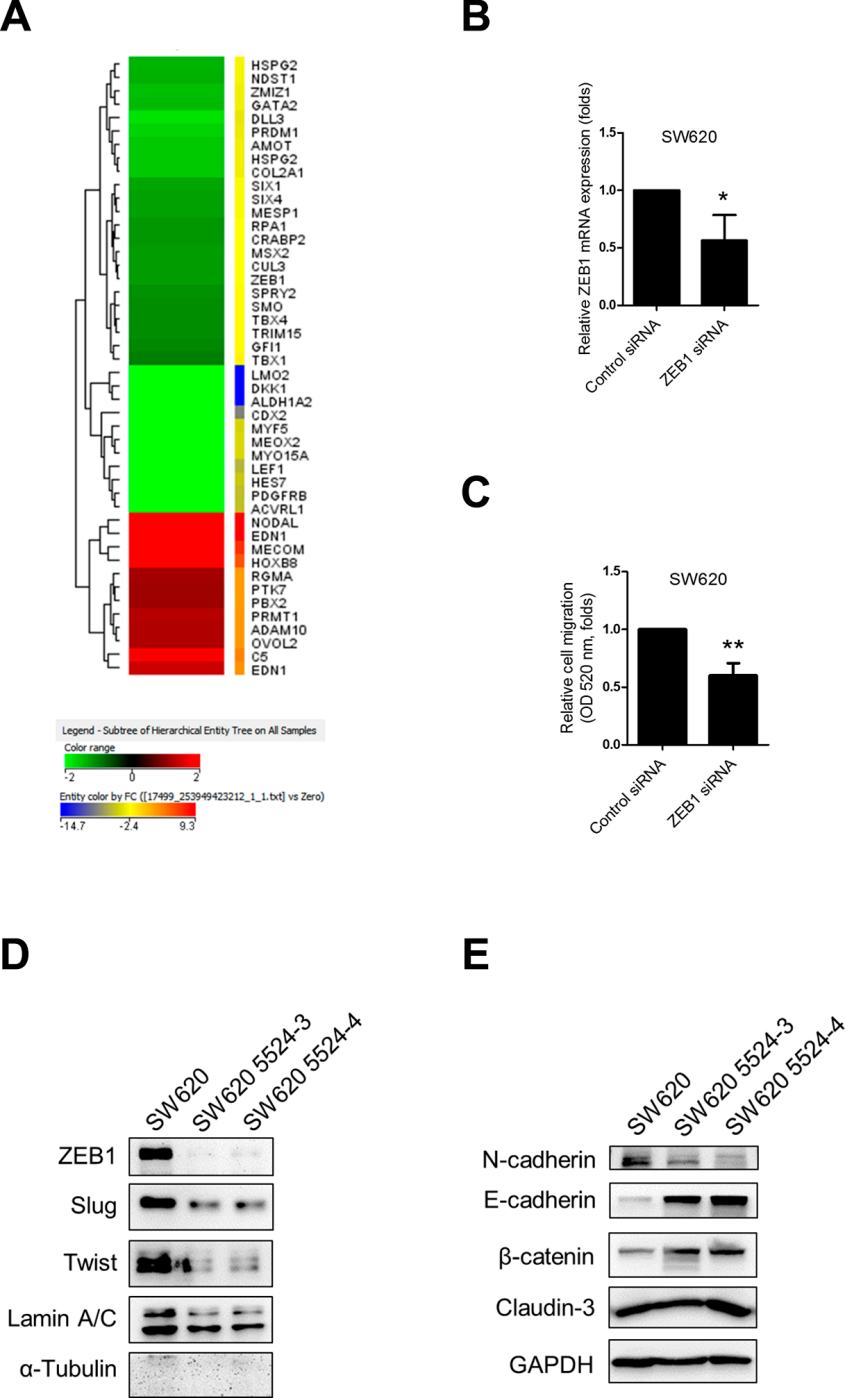
Suppression of NUCB-2 altered regulatory molecules of EMT (**A**) Heat map of genes with significant changes in Gene Ontology pathways contributing migration and invasion. (**B**) The effect of ZEB1 siRNA. The box plot showed mRNA expression of ZEB-1. (**C**) The box plot showed quantitative results of transwell migration assay. (**D**) Western blot assay showed regulatory molecules of EMT in nucleus and (**E**) in cytosol. Data represent mean ± SD. *p < 0.05; **p < 0.01.

**Figure 5:**
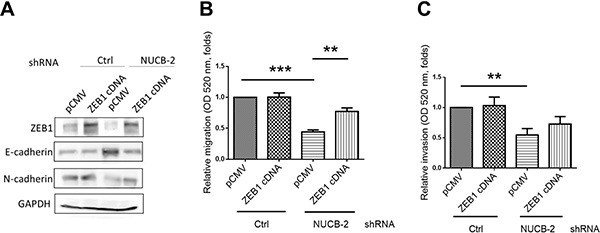
Suppression of NUCB-2 inhibited through regulation of ZEB-1 (**A**) The effect of ZEB-1 overexpression. Western blot assay showed the level of ZEB1, N-cadherin and E-cadherin. (**B**) ZEB-1 overexpression regained migration and (**C**) invasion ability of NUCB-2 knockdowned stable clones. Data represent mean ± SD. **p < 0.01; ***p< 0.001.

To further determine the regulatory mechanism, we performed microarray assay for analyzing gene expression pattern of nesfatin-1/NUCB-2 knockdowned SW620 and control SW620. Because nesfatin-1/NUCB-2 may enhance migration and invasion, the potential regulatory genes are shown in Figure 4A. Zinc finger E-box binding homeobox transcription factor 1 (ZEB1) is a transcription factor and a master regulator for EMT in several types of cancer [19, 20]. ZEB1 inhibition also led to reducing migration of SW620 (Figure 4B and 4C). In addition, low ZEB1 expression was detected in the nucleus of NUCB-2 knockdowned stable clones (Figure 4D). Twist and Slug are important transcription factors involved in EMT regulation [21]. The level of both molecules in NUCB-2 knockdowned SW620 was significantly lower than that in SW620, which was transfected with control vector (Figure 4D). The level of epithelial phenotype-related markers, including E-cadherin, β-catenin and Claudin-3, increased in NUCB-2 knockdowned stable clones (Figure 4E). In order to further determine the role of ZEB1, SW620 cells were transfected with the ZEB-1 expressing plasmid. ZEB-1 overexpression induced low E-cadherin and high N-cadherin expression in NUCB-2 knockdowned stable clones (Figure 5A). Furthermore, ZEB-1 overexpression regained migration ability in NUCB-2 knockdowned stable clones (Figure 5B and 5C). The results indicated NUCB-2 maintained mesenchymal properties in colon cancer cells through ZEB-1.

### NUCB-2 enhances migration and invasion in colon cancer through AMPK and TORC1 signaling pathways

**Figure 6:**
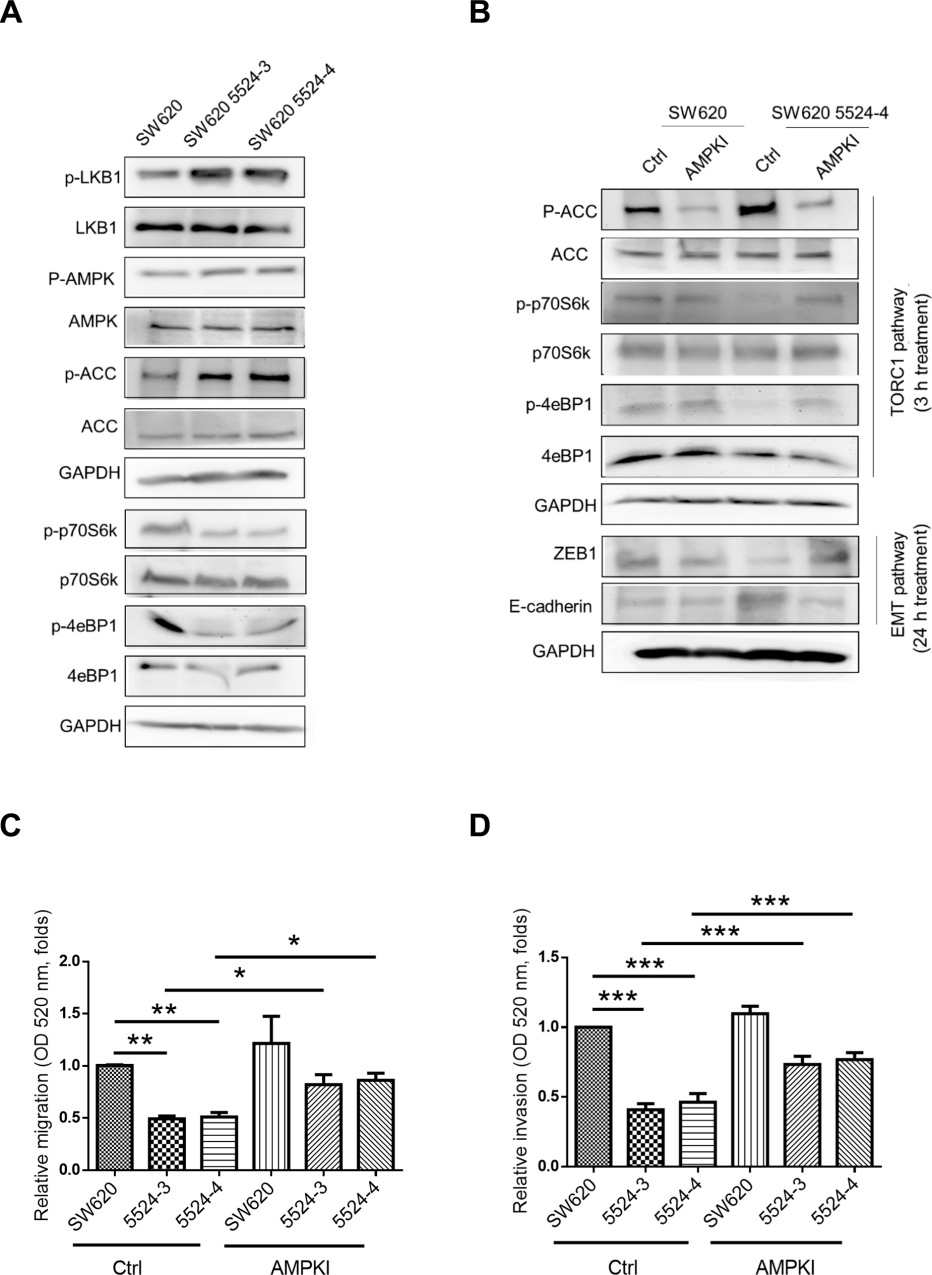
AMPK and TORC1 pathway were critical for regulating NUCB-2-mediated inhibition of migration and invasion (**A**) The effect of NUCB-2 suppression in AMPK and TORC1 pathways. (**B**) The effect of AMPK inhibitor. To determine the TORC1 and EMT pathways, protein was collected after 3 hours treatment and 24 hours treatment in NUCB-2 knockdowned stable clones respectively. (**C**) Treatment of AMPK inhibitor enhanced migration ability and (**D**) invasion ability of NUCB-2 knockdowned stable clones. Data represent mean ± SD. *p < 0.05; **p < 0.01; ***p < 0.001.

**Figure 7:**
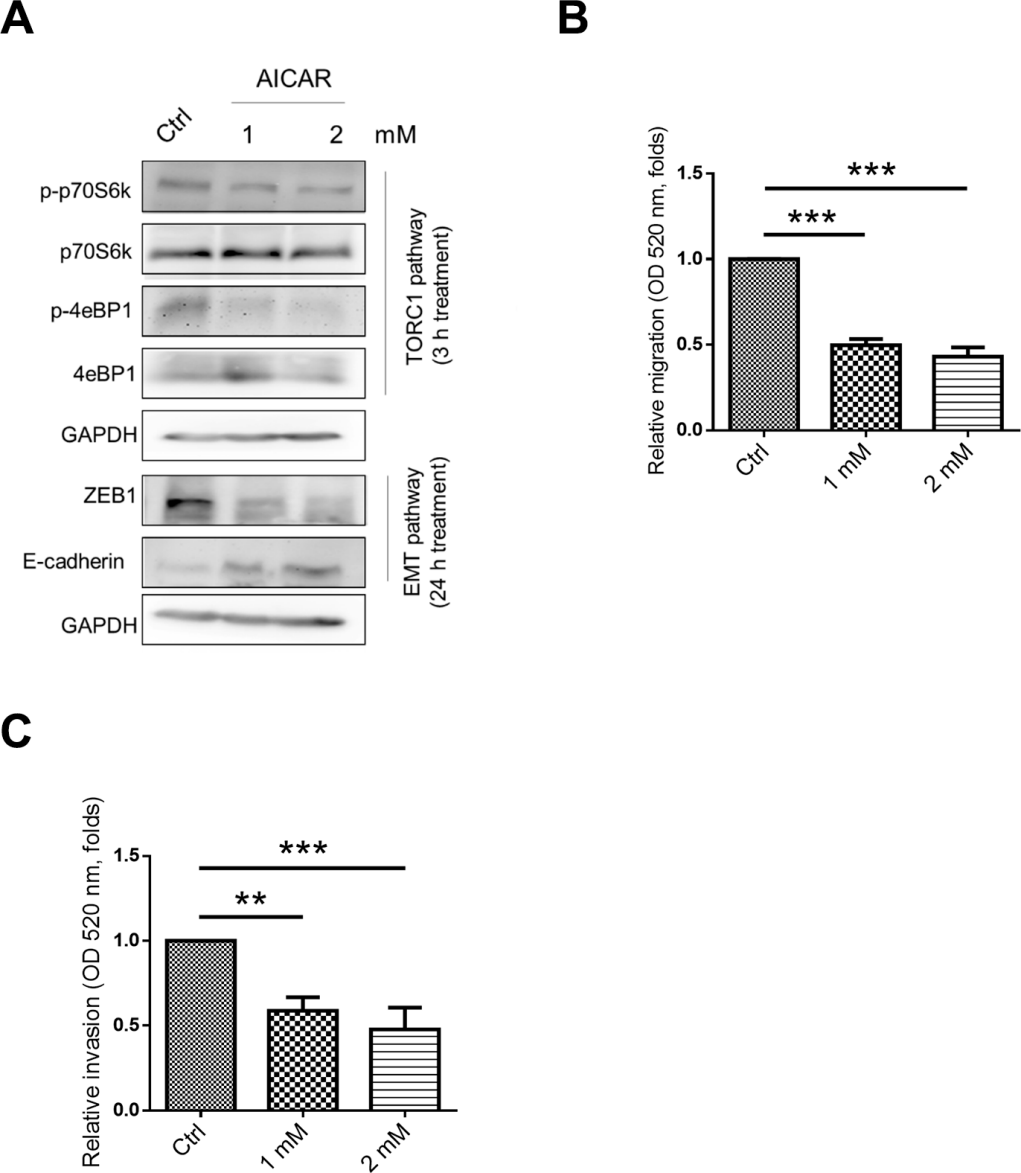
Activation of AMPK reduced migration and invasion of SW620 (**A**) The effect of AMPK activator AICAR in TORC1 and EMT pathways. (**B**) Treatment of AICAR inhibited migration and (**C**) invasion ability of SW620. Data represent mean ± SD. **p < 0.01; ***p < 0.001.

Nesfatin-1/NUCB-2 plays a role in energy homeostasis [22, 23]. AMPK and mTOR are also key regulators of energy homeostasis [24, 25]. In addition, nesfatin-1/NUCB-2 was demonstrated to trigger the mTOR and AMPK pathways [14, 17]. Because NUCB-2 signaling pathways were supposed to interact with AMPK/mTOR pathways, we investigated whether regulation of NUCB-2 and ZEB1 was dependent on liver kinase B1 (LKB1)/AMPK/mTOR pathways. In SW620, suppression of NUCB-2 increased phosphorylation of LKB1, AMPK and acetyl-CoA carboxylase (ACC) in AMPK pathways, and decreased phosphorylation of protein S6 kinase (S6K) and 4eBP1 in TORC1 pathways (Figure 6A). When AMPK inhibitor (compound C) was treated, the phosphorylation status of ACC, S6K, 4eBP1 was reversed. Furthermore, AMPK inhibitor enhanced ZEB-1 expression and reduced E-cadherin expression in NUCB-2 knockdowned stable clones (Figure 6B). The migration and invasion ability of NUCB-2 knockdowned clones was significantly enhanced after treatment of AMPK inhibitor (Figure 6C and 6D). To further investigate whether AMPK and TORC1 pathways involved in NUCB-2 regulated pathways in colon cancer, SW620 was treated with the AMPK activator AICAR. The effect of AICAR on the TORC1 pathway and EMT pathway was similar to the effect of NUCB-2 suppression (Figure 7A). AICAR treatment significantly inhibited migration and invasion also (Figure 7B and 7C). The results suggested that AMPK and TORC1 pathways are involved in NUCB-2 regulated EMT properties, migration and invasion in colon cancer.

### Suppression of NUCB-2 inhibits formation of tumor nodules in colon cancer-bearing mice

**Figure 8:**
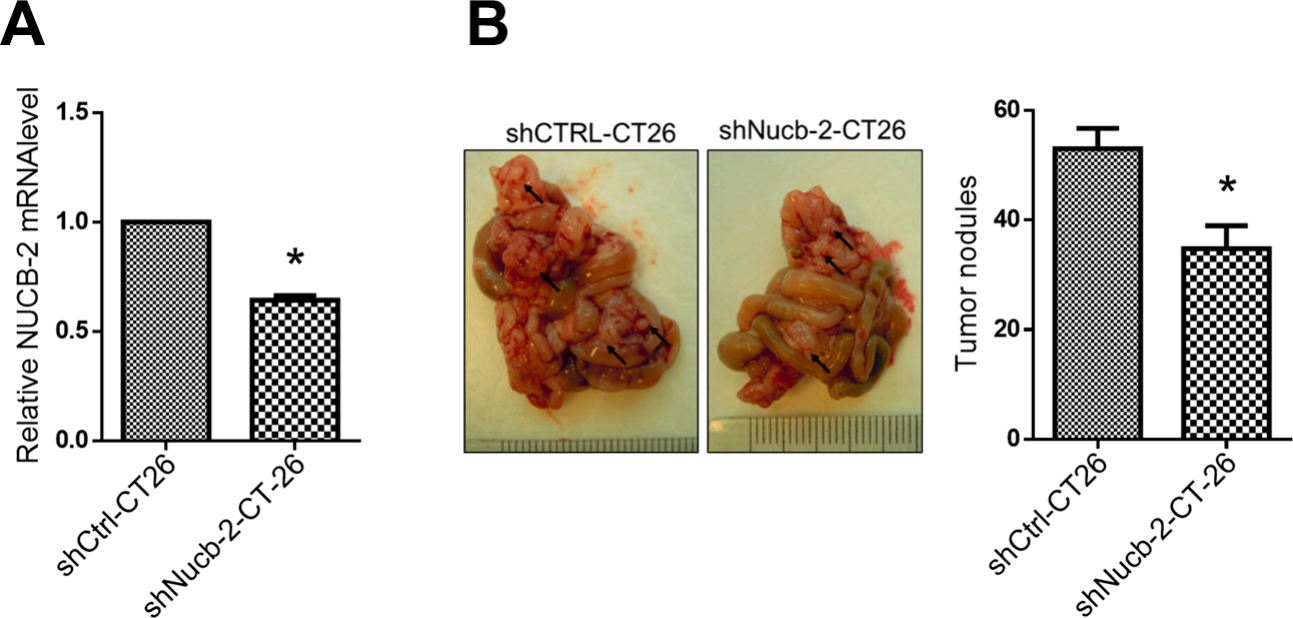
Suppression of NUCB-2 inhibits tumor formation in murine tumor model (**A**) Q-PCR assay showed that suppression of Nucb-2 in murine colon cancer cell CT-26. (**B**) The left panel showed the picture of tumor nodules. Black arrows indicated tumor nodules. The right panel showed number of tumor nodules in CT-26 tumor-bearing BALB/c mice (*n* = 5 in each group). Data represent mean ± SD. *p < 0.05.

To determine the role of NUCB-2 *in vivo*, CT-26, which is a murine colon cancer cell line, was transfected with NUCB-2 targeting shRNA or control shRNA. In Figure 8A, the level of NUCB-2 mRNA was suppressed after the shRNA transfection. The NUCB-2 shRNA stable clone or vector control CT-26 cells were injected intraperitoneally to BALB/c mice. The result showed NUCB-2 suppression also reduced the number of tumor nodules (Figure 8B). It indicates that NUCB-2 plays a critical role in colon cancer.

### NUCB-2 expression is associated with early metastasis in colon cancer

**Figure 9:**
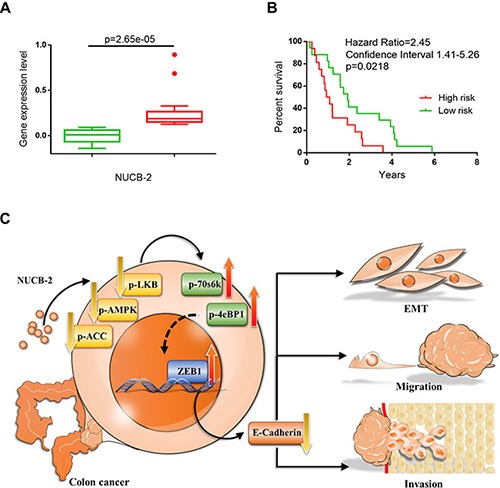
NUCB-2 enhanced metastasis in colon cancer (**A**) The box plot generated by SurvExpress program showed the expression levels of NUCB-2 and the *p*-value resulting from a *t*-test of the difference in online dataset (GEO accession: GSE28722). Low-risk (*n* = 17) and high-risk (*n* = 16) groups are shown in green and red respectively. (**B**) Kaplan-Meier time to metastasis curve using the SurvExpress program to analyze the sample from a GEO dataset (GSE28722). Low-risk (*n* = 17) and high-risk (*n* = 16) groups are shown in green and red respectively. (**C**) Scheme of proposed NUCB-2 mediated signaling pathways in colon cancer.

To investigate whether NUCB-2 expression was associated with tumor metastasis, the expression array (GEO accession: GSE28722) which contains thirty-three patients with metastatic colon cancer was analyzed through “time to metastasis” analysis in SurvExpress program. In Figure 9A, the high-risk group showed higher NUCB-2 expression than did the low-risk group. Kaplan-Meier analysis revealed the high-risk group had shorter time to metastasis (Figure 9B). The results confirm the role of NUCB-2 in metastatic colon cancer.

## DISCUSSION

Nasfatin-1/NUCB-2 shows diverse function in different types of tissues and cancers. In this study, we firstly demonstrated that nesfatin-1/NUCB-2 enhances cell migration and invasion in colon cancer. The expression of NUCB-2 in tumor regions is higher than that in non-tumor regions. Online dataset also indicated the NUCB-2 expression was associated with early metastasis. Therefore, the expression of NUCB-2 in tumor tissue may serve as a biomarker for predicting metastatic risk in colon cancer. On the other hand, abnormal serum or plasma level of NUCB-2 was detected in several types of diseases [26–28]. However, no significant difference was observed after analyzing the concentration of nesfatin-1 in serum of healthy donors and patients with colon cancer. It indicates that NUCB-2 overexpression in tumor does not result in elevation of nesfatin-1 in serum. The result may suggest NUCB-2 promotes tumor EMT via autocrine and paracrine pathways while the NUCB-2-derived-nesfatin-1 is not sufficient to elevate the serum concentration of nesfatin-1.

Nesfatin-1/NUCB-2 is distributed in multiple types of tissues, including adipose tissue, central neuron system, gastrointestinal system and reproductive organs [7]. Because each physiological function of each tissue is distinct, diverse pathways have been determined in different types of tissues. In colon cancer, the shape of SW620 was changed from spindle shape to rounded shape and migration and invasion ability was inhibited after suppression of NUCB-2. Thus, we hypothesized that NUCB-2 enhanced migration, invasion, and EMT pathways in colon cancer. Our microarray analysis showed the mRNA level of ZEB-1, which was a critical regulator of EMT genes, was associated with NUCB-2 expression. In addition, ZEB1 overexpression enhanced the migration and invasion ability in NUCB-2 knockdowned stable clones. To the best of our knowledge, this is the first study describing that ZEB-1 has a critical role in NUCB-2-mediated migration, invasion and EMT pathways in colon cancer. Twist and Slug are also important transcription factors, which regulate EMT phenotypes [28]. Although the mRNA level of Twist and Slug was not significantly changed in the NUCB-2 knockdowned stable clone in microarray analysis, suppression of NUCB-2 resulted in decreasing the protein level of Twist and Slug. The detailed mechanism needs further discussion.

The nesfatin-1/NUCB-2 signaling pathways are not well known currently. LKB1, AMPK and mTOR pathways regulate many important cellular processes, such as cell regulation of cell cycle, proliferation, energy homeostasis, migration and EMT in cancer [30–33]. Some studies have indicated the interaction between nesfatin-1/NUCB-2 and mTOR or AMPK pathways. In rat brain, nestafin-1 treatment enhanced phosphorylation of AMPK and TORC2 [17]. Gastric nesfatin-1/NUCB-2 is regulated by mTOR pathway under fasting and high fat diet condition [34]. Controversially, nesfatin-1 inhibits mTOR phosphorylation in dorsal motor nucleus of the vagus [35]. Nesfatin-1 treatment inhibits cell proliferation through decreasing mTOR phosphorylation and activating Ras homolog gene family, member A/Rho-associated protein kinase pathway in the ovarian epithelial carcinoma cell [14]. These studies suggest the nesfatin-1/NUCB-2 links to LKB1/AMPK/mTOR pathways in many types of tissues, although these signaling pathways induce quite different functions.

In breast and prostate cancer, AMPK reverses the mesenchymal phenotype to epithelial phenotype [36]. Treatment of metformin, which is an oral drug for diabetes mellitus type2, shows anti-cancer activity through inhibition of mTOR/AMPK and reverses EMT phenotypes [37, 38]. This evidence supports that the AMPK/mTOR pathway is important for EMT regulation. Here, we have demonstrated that the AMPK/TORC1 pathway is essential in nesfatin-1/NUCB-2-mediated migration, invasion, and EMT pathways. ZEB1 and E-cadherin expression is regulated by AMPK inhibitor (compound C) and activator (AICAR). It indicates the NUCB-2/AMPK/TORC1 pathways are upstream pathways of ZEB1 and EMT properties in colon cancer. A recent study indicates AMPK controls TORC1 pathways under nitrogen stress [39]. In addition, AMPK and TORC1 pathways sense the metabolic stress [40]. In this study, NUCB-2 knockdowned colon cancer cells showed activation in AMPK pathways and inhibition in TORC1 pathways. The energy stress might be a factor to control AMPK/TORC1 pathways in NUCB-2 knockdowned colon cancer cells. Furthermore, the ZEB1 expression is also regulated through mTOR signaling pathways in cholangiocarcinoma and pancreatic cancer cells [41, 42]. Therefore, we proposed AMPK might regulate ZEB1 expression through mTOR and TORC1 signaling pathways. However, it is currently unknown how a hypothalamic peptide nesfatin-1 or its precursor NUCB-2 triggers AMPK activation, or how AMPK/TORC1 regulates transcription factor ZEB1 and EMT-associated molecules. The detailed regulatory mechanism needs to be further investigated.

After determining the role of nesfatin-1/NUCB-2 in clinical tumor samples, serum samples, a cell line, an animal tumor model, and an online microarray dataset, our results indicated nesfatin-1/NUCB-2 was a potential biomarker for prediction of metastasis in colon cancer. In addition, we demonstrated nesfatin-1/NUCB-2 enhanced migration, invasion and mesenchymal phenotype in colon cancer through LKB1/AMPK/TORC1/ZEB1 pathways (Figure 9C). These findings may contribute to develop novel anti-cancer therapies against metastatic colon cancer in the future.

## MATERIALS AND METHODS

### Samples collection

One hundred and sixty adult patients (age > 18 years old) with colon cancer were collected from Kaohsiung Medical University Hospital. After obtaining informed consent, 10 ml of blood was drawn and then the serum was isolated and stored in aliquots in −80°C. The sera from one hundred and nineteen healthy donors were also collected after obtaining informed consent. Ten tumor samples were collected after surgery and were stored in −80°C. Approval for these studies was obtained from the Institutional Review Board of Kaohsiung Medical University Hospital.

### Materials and antibodies

AMPK inhibitor “Compound C” and AMPK activator AICAR were purchased from Sigma-Aldrich. Antibodies of AMPKa1 (1:1000), β-Catenin (1:2000), LKB1 (1:1000), Slug (1:500), ZEB1 (1:1000) and α-tubulin (1:2000) were obtained from Cell Signaling Technology; antibodies of Claudin-3 (1:1000), phospho-AMPKa1 (T172) (1:1000), phospho-LKB1 (S431) (1:500), acetyl-CoA carboxylase (ACC) (1:1000), phospho-ACC (S79) (1:500), vimentin (1:2000), and GAPDH (1:4000) were obtained from Millipore; antibodies of N-cadherin (1:1000), E-cadherin (1:2000), and Lamin A/C (1:2000) were obtained from BD Transduction Laboratories; antibody of nesfatin-1/NUCB-2 (1:1000) was obtained from R&D Systems; and antibody of Twist (1:500) was obtained from Sigma-Aldrich, respectively. The numbers in parentheses indicate the dilution fold of each antibody in western blot assay.

### Immunohistochemistry

The NUCB-2 expression was detected by anti-NUCB-2 antibody (R&D Systems, USA) in frozen-embed tumor samples. Briefly, frozen sections (5 μm) were stained with anti-NUCB-2 antibody (1:100) (R&D Systems) at 4°C overnight and then donkey anti-sheep antibody-Alexa 488 (1:200) at 37°C for 1 hour. DAPI (4′, 6-diamidino-2-phenylindol) staining was used for counterstain. The images were collected using a confocal microscope, LSM 700 (Carl Zeiss MicroImaging).

### ELISA

Human Nesfatin-1/Nucleobindin-2 ELISA kit (R&D Systems, #DY5949) was used for determining nesfatin-1 in serum samples or culture medium samples according to manufacturer's instruction.

### Cell culture

The human colon cancer cell lines, SW480 and SW620, were obtained from ATCC (Rockville, MD) and were cultured in Leibovitz's L-15 medium. Mouse colon cancer cell line CT-26 was also obtained from ATCC and cultured in RPMI-1640 medium. Both media were supplemented with 10% FBS and penicillin/streptomycin (100 U/0.1 mg/mL). All materials for cell culture were obtained from Invitrogen (Carlsbad, CA).

### Quantitative real-time PCR

Total RNA of cells was extracted from Trizol Reagent (Invitrogen). Complementary DNA (cDNA) of mRNA was performed through PrimeScript RT reagent Kit (Clontech). The primer of human NUCB-2 was 5′-TCTTGGAGCCAGATAGCTGG-3′ and 5′-AGCTTCTGAGCCTCCAGTTG-3′; human ZEB-1 is 5′-TATGAATGCCCAAACTGCAA-3′ and 5′-TGGT GATGCTGAAAGAGACG-3′; human Glyceraldehyde 3-phosphate dehydrogenase (GAPDH) is 5′-GAGTC AACGGATTTGGTCGT-3′ and 5′-TTGATTTTGGAGG GATCTCG-3′; mouse NUCB-2 is 5′-GGAGCCAAG TCCTGATCTCTAC-3′ and 5′-TTCAGACAGGCCAAG GTTTT-3′; mouse GAPDH is 5′-AACTTT GGCATTGT GGAAGG-3′ and 5′-ACACATTGGGGGTAGGAACA-3′. The level of mRNA was determined on StepOne Plus Real-Time PCR System (Applied Biosystems) using Fast SYBR Green Master Mix (Applied Biosystems). The relative gene expression was calculated by comparative delta-Ct method according to the formula: relative expression ratio = 2^−ΔΔCt^ = 2^− [ΔCt (ZEB1 siRNA) - ΔCt (control siRNA)]^, where ΔCt is equal to the Ct of ZEB1 minus the Ct of GAPDH.

### Western blot

Cells were lysed in radioimmunoprecipitation assay buffer (RIPA) buffer (Millipore) on ice for 30 minutes and the total cell lysate was collected after centrifugation at 4°C, 12000 × g for 15 minutes. For nuclear protein extraction, Nuclear Extract Kit (Active Motif) was used in the present study. Protein concentration was determined by BCA Protein Assay Kit (Novagen). Equivalent amount of protein was loaded and separated by sodium dodecyl sulfate−polyacrylamide gel electrophoresis (SDS−PAGE) (6−12%) and transferred to polyvinylidene difluoride membranes. The membrane was blocked in 5% non-fat dry milk for 1 hour and then incubated with each primary antibody overnight and peroxidase-conjugated secondary antibody for 1 hour. The results were detected using an enhanced chemiluminescence substrate (Millipore) on an imaging capture system (Alpha Innovation).

### Microarray

The RNA from NUCB-2 knockdowned SW620 cells and vector control SW620 were collected and performed microarray assay. The microarray experiment and data analysis were performed by Welgene Biotech (Taipei, Taiwan) using the Agilent SurePrint G3 Human V2 GE 8 × 60 K Microarray (Agilent Technologies, USA). The results were analyzed by Feature extraction 10.5.1.1 software (Agilent Technologies, USA), an image analysis and normalization software was used to quantify signal and background intensity for each feature, substantially normalized the data by rank-consistency-filtering LOWESS method.

### shRNA and stable clones

Short hairpin RNA (shRNA) targeting human NUCB-2, mouse NUCB-2 and a vector control construct (pLKO_AS2) were obtained from the National Core Facility for Manipulation of Gene Function by RNAi, miRNA, miRNA sponges, and CRISPR / Genomic Research Center, Academia Sinica, Taipei, Taiwan. SW620 cells were transfected with each shRNA plasmid using lipofectamine 2000 reagent (Invitrogen). For selection of stable clones, transfected cells were selected and maintained in medium containing 2 μg/ml puromycin.

### Cell proliferation assay

For scratch wound-healing assay, 5 × 10^4^ SW620 and NUCB-2 knockdowned SW620 cells were seeded into 96 well plate. The cell proliferation rate was determined by Premixed WST-1 Cell Proliferation Reagent (Clontech, CA) according to the manufacturer's instructions after 24 hours of incubation.

### Cell migration and invasion assay

For scratch wound-healing assay, 1 × 10^6^ SW620 cells were seeded into 24 well plate in 500 μl medium. The following day, a uniform scratch was made down the center of the well using a micropipette tip, followed by washing twice with 500 μl phosphate-buffered saline. Thirty hours after scratching, photographic imaging was performed using the Olympus 1 × 50 inverted microscopes with the NIS-Elements F 2.20 software (NIS-Elements; Melville, New York). For transwell migration and invasion assay, QCM™ 24-well Cell Migration Assay and Invasion System (Millipore), according to the manufacturer's instructions. Briefly, 2 × 10^5^ cells were seeded into 24 well insert in 300 μl serum free medium while 500 μl medium with 10% FBS was in lower chamber. Migration and invasion cells were evaluated by CyQuant GR Dye (Invitrogen) on a fluorometer (*FLx800* Microplate Fluorescence Readers, BioTek Instruments Inc, Winooski, VT) using a 480/520 nm filter.

### siRNA and cDNA transfection

ZEB1 siRNA (siRNA-mix from SMARTpool) and control siRNA were obtained from Dharmacon. Cells were transfected with siRNA (final concentration was 10 nM) by DharmaFECT transfection reagent 1 (Dharmacon) according to manufacturer's instruction. ZEB1 cDNA and control vector (pCMV) were obtained from OriGene and transfected by lipofectamine 2000 reagent (Invitrogen).

### Animal tumor model

The use of all the animals in this study was approved by the Animal Care and Use Committee at the Kaohsiung Medical University. Six-week-old male inbred mice (BALB/cByJNarl) were obtained from the National Laboratory Animal Center (Taiwan) and maintained in pathogen-free conditions. Mice were intraperitoneally injected with 1 × 10^6^ cells CT-26 cells in 200 microliter. Ten days after tumor cells injection, all mice were sacrificed and the tumor nodules were counted.

### Statistics

Differences between two independent groups were analyzed by student's *t*-test. For analyzing differences among three and four independent groups, one-way ANOVA test with Bonferroni's multiple comparison test was used. Significant difference (*p* < 0.05) between each group was considered. All experimental calculations were carried out using the program GraphPad Prism version 5.03 (GraphPad Software, San Diego, CA). The online biomarker validation tool SurvExpress (http://bioinformatica.mty.itesm.mx:8080/Biomatec/SurvivaX.jsp) was used for analyzing the correlation between NUCB-2 expression and metastasis in colon cancer [43]. The selected dataset (GEO accession number: GSE28722) was analyzed according to censored “time to metastasis” in Cox survival analysis in SurvExpress [44].
